# *In situ* preparation of monodispersed Ag/polyaniline/Fe_3_O_4_ nanoparticles via heterogeneous nucleation

**DOI:** 10.1186/1556-276X-8-309

**Published:** 2013-07-03

**Authors:** Longchun Bian, Lixia Bao, Jiliang Wang, Jingxin Lei

**Affiliations:** 1 School of Chemical Science and Technology, Yunnan University, Kunming 650091, People's Republic of China; 2 State Key Laboratory of Polymer Materials Engineering, Polymer Research Institute of Sichuan University, Chengdu 610065, People's Republic of China

**Keywords:** Nanoparticles, Ferromagnetic, *In situ* composites, Polyaniline, Self-assembly

## Abstract

Acrylic acid and styrene were polymerized onto monodispersed Fe_3_O_4_ nanoparticles using a grafting copolymerization method. Aniline molecules were then bonded onto the Fe_3_O_4_ nanoparticles by electrostatic self-assembly and further polymerized to obtain uniform polyaniline/Fe_3_O_4_ (PANI/Fe_3_O_4_) nanoparticles (approximately 35 nm). Finally, monodispersed Ag/PANI/Fe_3_O_4_ nanoparticles were prepared by an *in situ* reduction reaction between emeraldine PANI and silver nitrate. Fourier transform infrared and UV-visible spectrometers and a transmission electron microscope were used to characterize both the chemical structure and the morphology of the resulting nanoparticles.

## Background

Researches regarding polymer-metal and polymer-inorganic multicomponent hybrid composites such as polyaniline/silver (PANI/Ag), poly(ethylene oxide)/aurum (PEO/Au), PANI/Fe_3_O_4_, etc. have attracted much attention during the last two decades due to their large potential applications in the fields of electromagnetic interference (EMI) shielding [1-3], energy storage devices [4-6], catalysis [7-9], and sensors [10-14]. These hybrid composites show better chemical and physical properties than bulk materials.

Among various polymers, PANI as a versatile conducting polymer is usually selected to compound with noble metals or inorganic particles owing to its easy preparation, anticorrosion, and the low cost of raw material. Recently, Kamchi et al. [3] have elaborated serials of camphor-doped PANI/FeNi nanoparticle-based EMI shielding composites. The maximum conductivity value of 10^4^ S m^-1^ and the shielding effectiveness (SE) of 90 dB of the prepared multilayer composites have been detected over the frequency band of 8 to 18 GHz. Leyva et al. [15] have successfully synthesized kinds of hybrid organic/inorganic PANI-Ag powders via *in situ* chemical oxidation of PANI-emeraldine base by the capped Ag^+^ on the PANI-emeraldine base surface. The electrical direct current conductivity of the resulting PANI-Ag reaches 3.5 × 10^3^ S m^-1^ at room temperature, showing a good conductivity. Moreover, Shukla et al. [16] have also prepared homogeneous PANI-Ag core-shell nanorods synthesized via a mild photolysis-initiated ultraviolet radiation. The core-shell nanorods display a strong blueshift in the UV-visible (UV–vis) absorption spectrum and have instant application as a highly sensitive hydrazine and hydrogen peroxide sensor. However, the EMI shielding properties have not been studied. In addition, relevant PANI-based nanowires, nanorods, and core-shell nanoparticle EMI composites have been successfully prepared elsewhere [17-20]. Actually, many researches have been done to improve both the EMI SE and the cost performance by enhancing the conductivity and lowering the magnetic loss. Unfortunately, most of the developed hybrid EMI shielding materials are binary composites comprised of polymer/metal, polymer/inorganic, or metal/inorganic. These materials still suffer disadvantages of low EMI SE, limited shielding frequency range, high density, and high cost. Furthermore, from the angle of the crystal growth dynamics, most of the developed binary composites are simple blends or epitaxial blends. In the *in situ* preparation process of the second layer of the binary hybrid nanoparticles or nanocomposites, an obvious contradiction between the formation of more homogeneous nucleations and the heterogeneous nucleation and epitaxial growth of the second layer should be firstly solved. Usually, the formation of more homogeneous nucleations implies the formation of more separated nanoparticles, i.e., low efficiency to obtain the monodispersed binary nanoparticles. A few papers report the synthesis method to prepare monodispersed binary nanoparticles such as Ag or Au/Fe_3_O_4_ nanoparticles [21-25]. Supermagnetic and conductive properties of the performed monodispersed nanoparticles have also been particularly studied. However, these methods are only facilitated to prepare metal/inorganic binary nanoparticles.

To the best of our knowledge, almost no papers regarding synthesis and EMI shielding application of monodispersed multilayer nanoparticles have been reported. Consequently, studies about the synthesis and the application evaluation of PANI multicomponent nanocomposites such as PANI/metal/inorganic, metal/PANI/inorganic, or metal/inorganic/PANI ternary nanocomposites, especially the monodispersed nanocomposites, are necessary since noble metals, e.g., Au and Ag, usually own high electronic conductivity and PANI possesses both a low density and a considerable conductivity. To achieve this aim, the following two points should be considered prior to the preparation: (1) Mild reaction conditions are necessary to obtain the monodispersed nanoparticles. (2) A suitable solvent (polar or non-polar) is also an important factor to both the nucleation and the growth of the nanoparticles. Herein, we have prepared a monodispersed Ag/PANI/Fe_3_O_4_ ternary nanoparticle via a typical grafting copolymerization, an electrostatic self-assembly, and an *in situ* reduction of Ag^+^ on the surface of the PANI-emeraldine base polymeric chains.

## Methods

**Figure 1:**
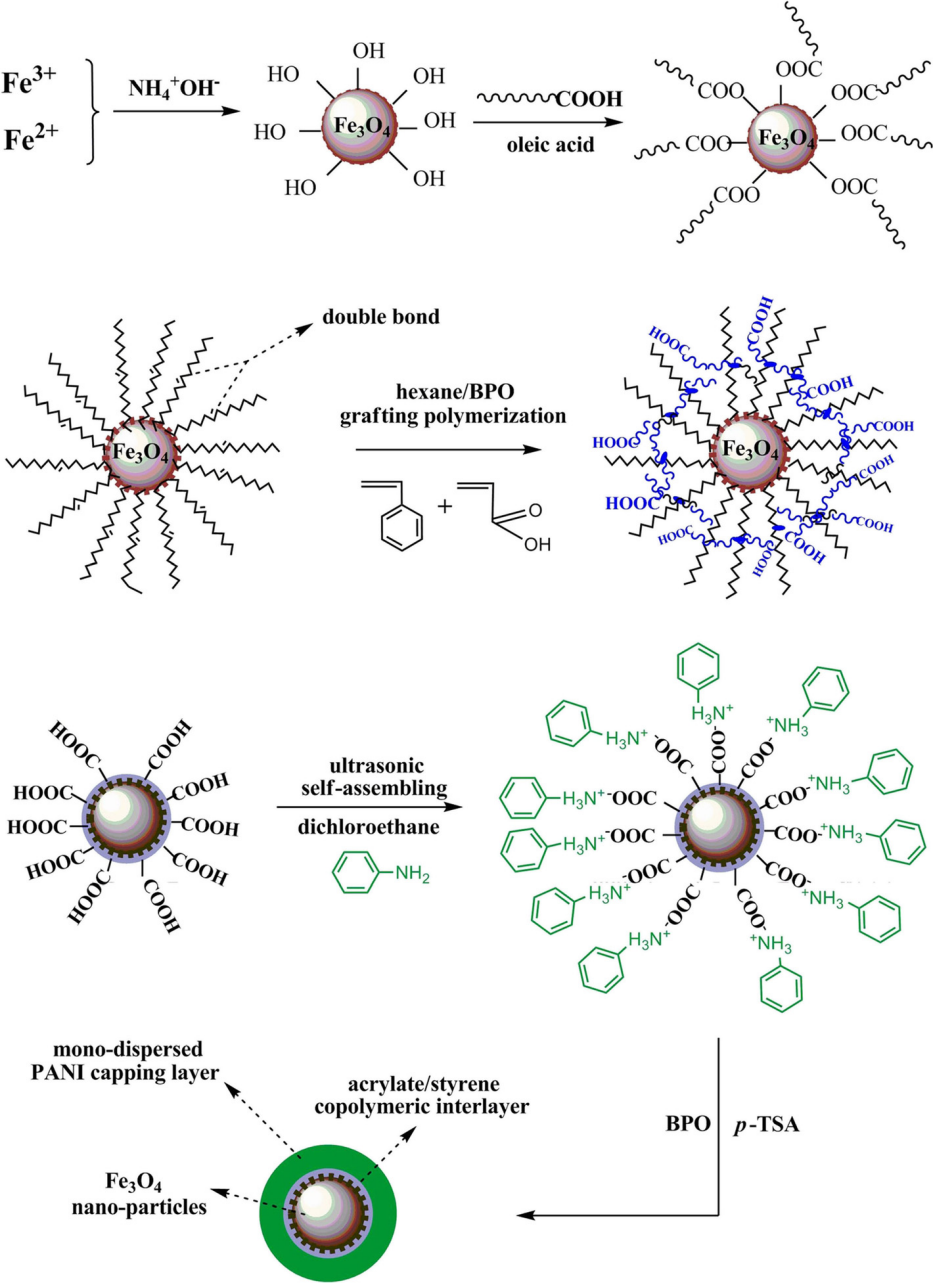
**Synthesis route of PANI/Fe**_**3**_**O**_**4 **_**nanoparticles.**

**Figure 2:**
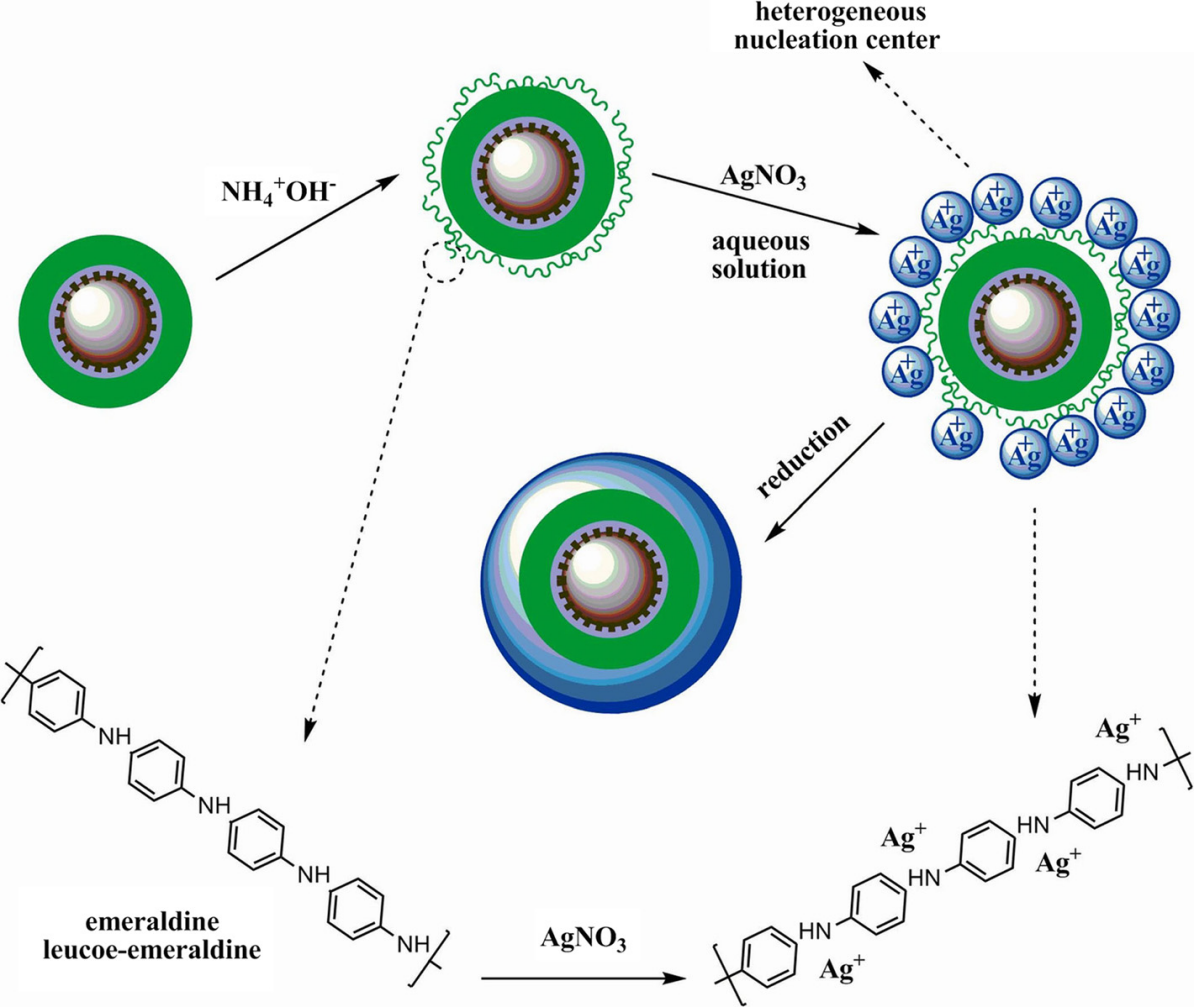
**Schematic synthesis procedure of Ag/PANI/Fe**_**3**_**O**_**4 **_**monodispersed nanoparticles.**

The copolymer-capped monodispersed Fe_3_O_4_ nanoparticles were firstly obtained as follows: 7.85 g of FeCl_3_ · 6H_2_O and 2.93 g of FeCl_2_ · 4H_2_O were dissolved in 200 mL distilled water at 80°C; 22 mL of 7.1 mol L^-1^ NH_4_OH was then quickly added into under ultrasonication, and the ultrasonication was maintained for 30 min. After another 1 h, diluted HCl was added to neutralize the resulting solution. Then 5 mL oleic acid was added dropwise over a period of 30 min. The resulting Fe_3_O_4_ nanoparticles were dissolved in hexane, and the concentration of 1 g L^-1^ Fe_3_O_4_ nanoparticle magnetic fluid was obtained. After that, 150 mL of 1 g L^-1^ magnetic fluid was diluted with 150 mL hexane and then added into a four-neck flask at 68°C; 10 wt.% of the mixed solution of 0.04 g of styrene, 0.04 g of acrylic acid, 0.03 g of benzoyl peroxide (BPO), and 15 mL hexane was quickly added into the flask, and the polymerization was maintained for 30 min. The residual 90 wt.% solution was added into the flask dropwise over a period of 2 h. After 8 h, the resulting magnetic fluid was centrifuged, and the obtained brown solid was then washed with acetone several times to remove homogeneous polymers. After that, ANi was added into the resulting copolymer-capped Fe_3_O_4_ solution under ultrasonication to insure that *N* atoms of ANi were effectively bonded with carboxyl groups of AA capped on the Fe_3_O_4_ nanoparticles. BPO and *p*-toluenesulfonic acid (*p*-TSA) were added into the ANi/Fe_3_O_4_ magnetic fluid dropwise to initiate the polymerization. The synthesis route of monodispersed PANI/Fe_3_O_4_ nanoparticles is shown in Figure 1. The prepared PANI/Fe_3_O_4_ nanoparticles were redispersed into deionized water. AgNO_3_ solution was then quickly added into the suspension under ultrasonication. The *in situ* reduction reaction between *N* atoms of emeraldine PANI and Ag^+^ was mildly continued with mechanical stirring for 48 h at room temperature to obtain the monodispersed Ag/PANI/Fe_3_O_4_ nanoparticles (Figure 2).

Fourier transform infrared (FTIR, Nicolet 560, Nicolet Instruments, Inc., Madison, WI, USA) and UV–vis (Shimadzu UV-2100, Shimadzu Corporation, Kyoto, Japan) spectrometers have been used to monitor the preparation process of the nanoparticles. The morphology of the prepared PANI/Fe_3_O_4_ binary nanoparticles and Ag/PANI/Fe_3_O_4_ ternary nanoparticles has also been extensively evaluated using a JEOL JEM-2100 electron microscope (JEOL Ltd., Akishima-shi, Japan) operating at an accelerating voltage of 200 kV.

## Results and discussion

**Figure 3:**
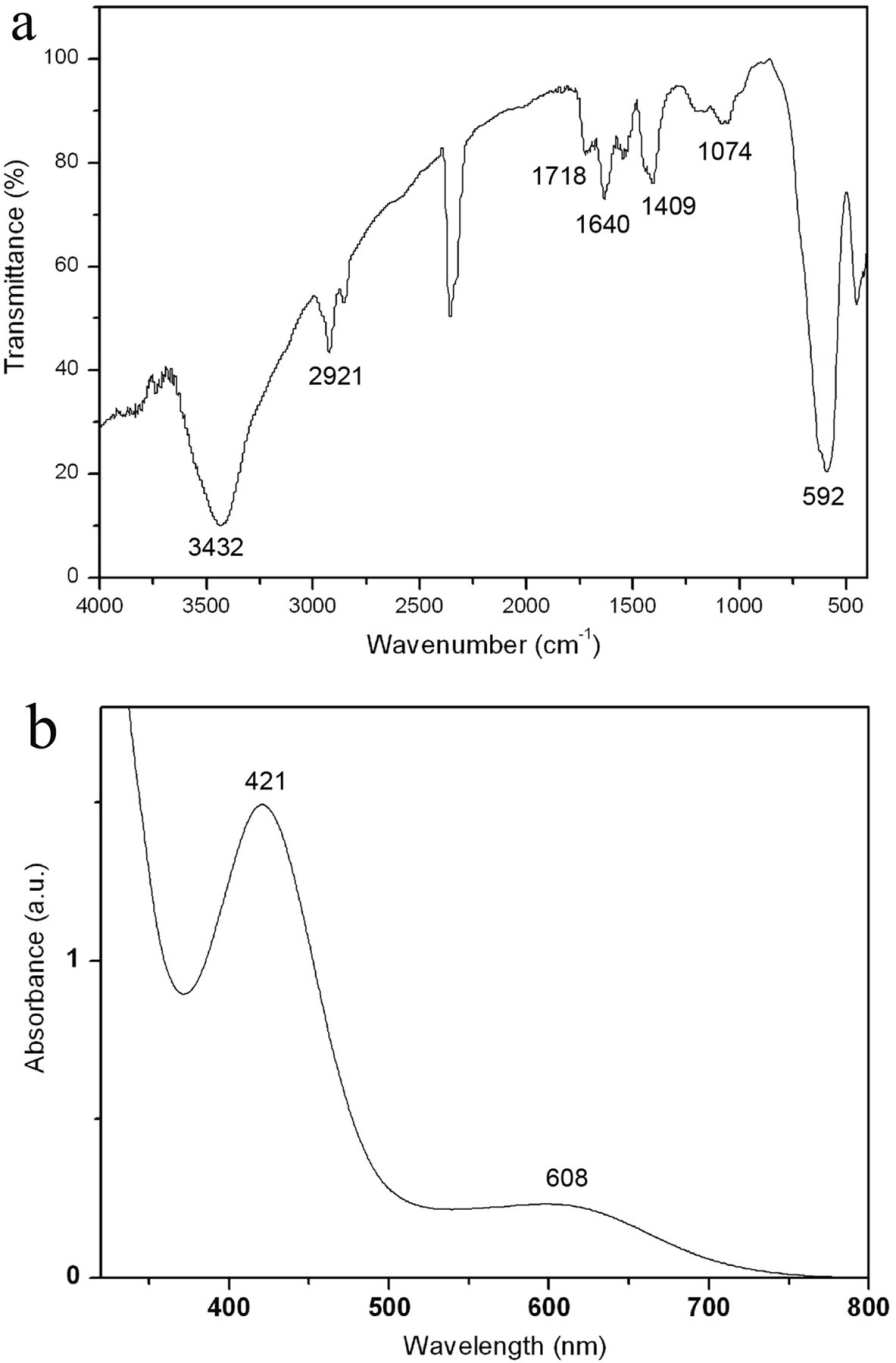
**Spectra of (a) FTIR of cografting polymer-coated Fe**_**3**_**O**_**4 **_**and (b) UV–vis of PANI/Fe**_**3**_**O**_**4 **_**nanoparticles.**

Figure 3a,b shows the FTIR spectrum of the grafting copolymer-coated monodispersed Fe_3_O_4_ nanoparticles and the UV–vis spectrum of the PANI/Fe_3_O_4_ nanoparticles, respectively. As shown in Figure 3a, absorption peaks at around 637, 592, and 451 cm^-1^ corresponding to the Fe-O stretching are observed. The characteristic peaks of Fe-O of the copolymer-capped Fe_3_O_4_ are found to shift towards the short-wavenumber region (blueshift) in comparison with those of typical uncapped Fe_3_O_4_ particles. Furthermore, obvious peaks at around 1,640, 1,550, and 3,030 cm^-1^ are detected which are characteristic peaks of -C = C- stretching and = C-H vibration of benzene ring, respectively. In addition, absorption peaks at about 3,432, 1,718, and 1,074 cm^-1^ deriving from -OH, -C = O, and -C-O- vibrations of -COOH, respectively, are also observed. Moreover, characteristic peaks at about 2,921 and 1,409 cm^-1^ originating from -CH_3_ of oleic acid chains are detected as well. The FTIR results apparently indicate that Fe_3_O_4_ nanoparticles are successfully capped by the AA/St grafting copolymers. After the grafting copolymerization, the copolymer-coated Fe_3_O_4_ nanoparticles can spontaneously precipitate rather than dissolve in hexane. This phenomenon can also confirm the formation of the copolymer-capped Fe_3_O_4_ nanoparticles to some extent because of the bad miscibility between the non-polar hexane and the copolymers. It is shown in Figure 3b that characteristic peaks of a typical doped PANI in the scales of <350, 400 to 500, and 500 to 700 nm corresponding to *π*-π*, polaron-π* (*trans*), and polaron or bipolaron transitions, respectively, are detected [10,26], revealing the achievement of the PANI-capped Fe_3_O_4_ nanoparticles. However, there is an obvious redshift of the characteristic absorption peaks (421 and 608 nm) in comparison with traditional inorganic acid-doped PANI, which is the comprehensive result of *p*-TSA and macromolecular poly(acrylic acid)-doped PANI. The obtained PANI chains probably form more extended conformations.

**Figure 4:**
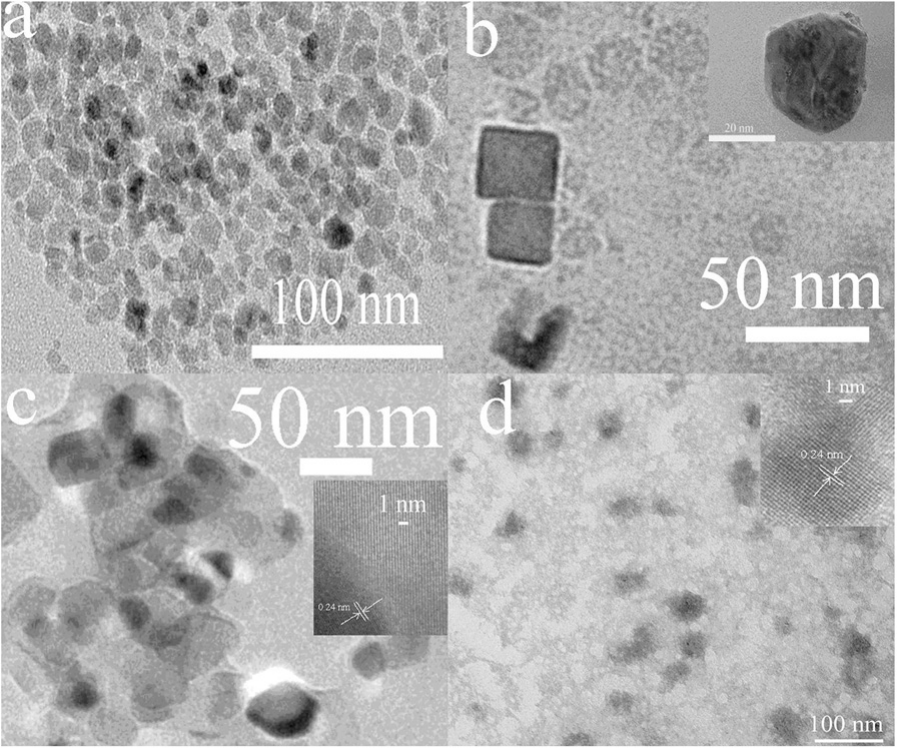
**TEM images of (a) oleic acid-coated Fe**_**3**_**O**_**4**_**, (b) PANI-capped PANI/Fe**_**3**_**O**_**4**_**, and (c, d) Ag/PANI/Fe**_**3**_**O**_**4 **_**monodispersed nanoparticles.** The insets in (b) and (c, d) show HR-TEM images of PANI/Fe_3_O_4_ and the lattice of Ag/PANI/Fe_3_O_4_ nanoparticles, respectively.

Figure 4a illustrates the morphology of oleic acid-coated Fe_3_O_4_ nanoparticles prepared by the coprecipitation method. It can be seen that Fe_3_O_4_ pre-spheral nanoparticles with a size range of 5 to 15 nm are found evenly dispersed into the transmission electron microscopy (TEM) view and that the size distribution of the Fe_3_O_4_ nanoparticles is relatively narrow. Most of the Fe_3_O_4_ nanoparticles own a size near 10 nm, and the distance between two near particles is only in the scale of 1 to 2 nm, showing a pre-monodispersity. After capping with the *in situ* polymerized PANI, both the size range and the shape of the Fe_3_O_4_ nanoparticles are changed (see Figure 4b). The enlarged view of the pre-spheral PANI/Fe_3_O_4_ nanoparticle is also shown in the right top inset of Figure 4b; the size range of the achieved PANI/Fe_3_O_4_ nanoparticles increases to 20 ~ 25 nm, and some cubic-like PANI/Fe_3_O_4_ nanoparticles with a size range of 25 to 35 nm are also detected. In addition, the distance between two neighboring nanoparticles enhances to 3 to 5 nm. The above phenomena reveal that the shape (pre-spheral) of the Fe_3_O_4_ nanoparticles is almost unchanged with the oxidation polymerization of ANI and that the thickness of the layer of PANI capped onto the monodispersed Fe_3_O_4_ nanoparticles is about 10 to 20 nm, which is nearly equivalent to the thickness of the Fe_3_O_4_ cores. Moreover, the PANI/Fe_3_O_4_ nanoparticles also maintain the monodispersity like pure Fe_3_O_4_ nanoparticles. Almost no aggregating PANI/Fe_3_O_4_ nanoparticles have been detected in the TEM view. The right top inset of Figure 4b also shows that the PANI layer is composed of many smaller irregular PANI particles with a size range of approximately 2 nm, implying that heterogeneous nucleation and epitaxial growth of PANI rather than homogeneous nucleation and formation of separated PANI particles are dominant during the mild oxidation polymerization of ANI, and this is the crucial factor for successfully preparing monodispersed PANI/Fe_3_O_4_ nanoparticles.

Figure 4c,d shows the morphology of the Ag/PANI/Fe_3_O_4_ nanoparticles at different TEM views. In the case of Figure 4c, many gray, even dark, pre-spheral particles with a size range of 30 to 50 nm are detected. The color of the nanoparticles is apparently darker than that of PANI/Fe_3_O_4_ nanoparticles, demonstrating the possible formation of Ag/PANI/Fe_3_O_4_ nanoparticles. The TEM morphology of the Ag/PANI/Fe_3_O_4_ nanoparticles at another view (different district) can be also used to confirm this assumption even if the background of the TEM graph is coarse (see Figure 4d) because the color of the observed nanoparticles is almost dark, originating from the existence of heavy metal Ag. Figure 4d also reveals that the obtained Ag/PANI/Fe_3_O_4_ nanoparticles are still monodisperse and that the distance between two particles further increases in comparison with the PANI/Fe_3_O_4_ nanoparticles. Furthermore, a high-resolution TEM (HR-TEM) technique is also performed, and the HR-TEM images are shown on the right top inset of Figure 4c,d. As can be seen from the HR-TEM images, obvious lattices originating from Ag are observed. In the lattice structures, the *d*-space of the (111) lattice is about 0.24 nm, which is the characteristic of Ag [22-24]. In addition, the HR-TEM images show that there are transitional layers between the lattice fringes of Ag and the PANI/Fe_3_O_4_ nanoparticles. Ag^+^ in the silver nitrate solution can be *in situ* reduced to gradually form Ag particles and coat onto PANI layers by interacting with the *N* atoms of emeraldine PANI polymer chains, while separated Ag particles cannot be observed at all due to the lack of an effective reducer in the solution, i.e., the homogeneous nucleation of Ag particles is thoroughly restrained. This is the reason why the monodispersed Ag/PANI/Fe_3_O_4_ nanoparticles can be obtained by the mild reduction reaction.

## Conclusions

In summary, monodispersed Ag/PANI/Fe_3_O_4_ ternary nanoparticles with an average size of approximately 50 nm can be successfully obtained by incorporating grafting copolymerization, electrostatic self-assembly, and mild reduction reaction method between the *N* atoms of PANI chains and the silver cations of silver nitrate solution. The control of heterogeneous nucleation and corresponding epitaxial growth of both PANI and Ag is crucial to prepare monodispersed Ag/PANI/Fe_3_O_4_ nanoparticles. The obtained monodispersed Ag/PANI/Fe_3_O_4_ nanoparticles have large potential applications in the fields of EMI shielding materials, biology, catalysis, etc.

## Competing interests

The authors declare that they have no competing interests.

## Authors’ contributions

LCB and LXB carried out the preparation and main characterization of different samples and drafted the manuscript. JLW and JXL participated in the design of the study and the manuscript modification. All authors read and approved the final manuscript.
